# Intergenerational reproduction and self-assessed mental health in adulthood in China

**DOI:** 10.1016/j.ssmph.2024.101645

**Published:** 2024-02-24

**Authors:** Xueqing Zhang, Gerry Veenstra

**Affiliations:** Department of Sociology, The University of British Columbia, Canada

**Keywords:** China, Health inequalities, Intergenerational reproduction, Self-reported childhood social class, Socioeconomic status, Self-assessed mental health

## Abstract

Physical and mental health disparities by socioeconomic status in China are well documented but the effects of the intergenerational reproduction in socioeconomic status on adult mental health have received little attention to date. We utilized cross-sectional data from the 2017 Chinese General Social Survey to examine the significance of intergenerational socioeconomic reproduction for differences in self-assessed mental health in a national sample of Chinese adults between the ages of 23 and 65. We documented substantial elasticities between the socioeconomic status of the survey respondents and their parents: father's education, mother's education and childhood social class were all associated with both respondent education and respondent household income. We also found that associations between parental socioeconomic status and their adult children's self-assessed mental health were partly explained by the children's own socioeconomic status. However, these pathways were noticeably moderated by age cohort. Among younger people, associations between parental socioeconomic status and mental health were mostly explained by educational attainment whereas among older people associations between parental socioeconomic status and mental health were mostly explained by household income. In general, parental socioeconomic status appear to have a greater influence on the mental health of people who grew up after the Chinese economic reform of the 1970s.

## 1 Introduction

Strong associations between socioeconomic status (SES; comprised of one or more of education, income and occupation) and health-related factors in China have been well documented (Huang & Yin, 2013; Wang, 2012; Wang & Geng, 2019; Zhang et al., 2009). For example, Zhang et al. (2009) determined that education and income are both associated with women's healthcare-seeking behaviors. Wang and Geng (2019) found that the International Socio-Economic Index (ISEI) is associated with physical health in adulthood, with lifestyle playing an important mediating role, and Huang and Yin (2013) found that Chinese women with higher education and more presitigious occupations have lower risks of disease and other health problems. In regards to psychological wellbeing, strong connections between socioeconomic status and mental health have been thoroughly documented in many national contexts (Adler & Newman, 2002; Link & Jo, 1995). Chinese researchers in particular have found that family socioeconomic status has a significant positive effect on teenagers' mental health (Yang et al., 2022), especially for girls (Li et al., 2022), and that, in the adult population, self-assessed social class and family income both have positive effects on mental health (Wang, 2022). In China, as in many other national contexts, socioeconomic status and mental health appear to be highly interconnected.

A plethora of studies documenting socioeconomic inequalities in both physical and mental health in China notwithstanding, there has been little research focused explicitly on the implications of intergenerational reproduction for adult physical health (but see Zhang & Veenstra, 2022) and no research focused on intergenerational reproduction and mental health in particular. In other words, health researchers in China have yet to illuminate the degree to which intergenerational production, processes by which wealthy and well-educated parents reproduce their socioeconomic standing in their children, are implicated in socioeconomic inequalities in physical and mental health. The intergenerational reproduction part of these complex intergenerational processes has been previously confirmed. The Development Research Group at the World Bank estimates that 49% of Chinese children born to parents in the top quartile of years of schooling will end up in the top quartile of years of schooling themselves, what economists refer to as intergenerational elasticities in education (Mobility Global Database on Intergenerational, 2018; Narayan et al., 2018). The group also estimates that the correlation between parent and offspring incomes in China is as high as 0.40 (Mobility Global Database on Intergenerational, 2018; Narayan et al., 2018). Moreover, the intergenerational income elasticity rose from 0.39 for the 1970–1980 birth cohort to 0.44 for the 1981–1988 birth cohort (Fan et al., 2019), suggesting that intergenerational elasticities in income in China may be rising over time.

Three processes have been proffered for how parental and personal socioeconomic resources might together and distinctly impact mental health in adulthood: social trajectory, cumulative exposure, and sensitive period (Berkman, 2009). According to the “social trajectory” scenario, parental socioeconomic resources facilitate acquisition of personal socioeconomic resources which in turn influence adult mental health. That is, the effects of parental socioeconomic resources on respondents' mental health are mediated by respondents' own socioeconomic resources. In support of this scenario, a study in the U.S. found that no effects of parental education on respondents' depressive symptoms persisted after controlling for respondents' socioeconomic factors (Quesnel-Vallée & Taylor, 2012). In South Korea, Jeong and Veenstra (2017) found that parental education was associated with depression before but not after controlling for the respondent's own education and income. The “cumulative exposure” scenario suggests that risks accumulate additively and, therefore, parental and personal socioeconomic resources shape adult mental health independently of one another. And, finally, the “sensitive period” scenario suggests that early life factors (e.g., parental socioeconomic resources in childhood) predict adult mental health irrespective of socioeconomic resources in adulthood. Research indicates that the latter scenarios also have some explanatory power. A Finnish study found that parental socioeconomic resources in youth were impactful for mental health in adult life, associations that were only slightly explained by personal socioeconomic resources (Harper et al., 2002; Kestilä et al., 2006). A U.S study found that both parents' education attainment were correlated with respondents' happiness but that only father's education remained significant after controlling for respondents' income (Nikolaev & Burns, 2014). Also, a Canadian study identified a correlation between low maternal education and risk of serious depression in young adulthood that was independent of respondents' education and income (Park et al., 2013).

According to life course theory, “individuals construct their life trajectories through choices and actions under the opportunities and constraints of the historical and social environment” (Elder et al., 2003; see also (Ben-Shlomo et al., 2016; Kuh et al., 2003; Pearlin & Bierman, 2013; Pearlin & McKeanSkaff, 1996). A person's health is not entirely representative of individual-level factors such as their socioeconomic resources, health behaviors and access to medical services but is also shaped by the historical and social contexts within which they live. In addition, changes in societal conditions can condition the effects of some individual-level factors on individual-level health. Over the past century, China has experienced fundamental transformations to the nature of social stratification in the country (Bian, 2002; Xie & Zhang, 2019). For example, the Chinese Communist Revolution, whose pinnacle was the establishment of the People's Republic of China in 1949, and the Cultural Revolution which spanned from 1966 to 1976, elevated the social status of children from the peasant, worker and revolutionary cadre classes while disadvantaging those from privileged classes (Murphree, 2010; Xie & Zhang, 2019). In addition, beginning in the late 1970s, the Chinese economic reform facilitated a shift of the nation's economic system from being primarily centrally planned to being primarily market-oriented. This economic reform resulted in the emergence of a significant number of people known as the “new business elites” (Pearson, 1997). Given these historical changes, we stratify our study participants by age, distinguishing between survey respondents born before 1972, the beginning of a period of profound social transformation in China, and those born in 1972 or later. This distinction allows us to obtain insight into the relevance of societal changes forthcoming from the Cultural Revolution and the Chinese economic reform on the mental health effects of intergenerational reproduction in China.

In light of the above, we pursue the following research questions utilizing a nationally representative survey dataset of Chinese adults: 1) Are parental socioeconomic resources associated with their adult children's socioeconomic resources? 2) Are parental socioeconomic resources associated with feelings of anxiety or depression in their adult children? 3) If parental socioeconomic resources are associated with feelings of anxiety or depression in their adult children, do the socioeconomic resources of the children explain the associations? 4) Finally, do these proposed pathways manifest themselves differently for older and younger Chinese people?

## 2 Methods

Our study utilizes data from the 2017 Chinese General Social Survey. The Chinese General Social Survey (CGSS) was initiated in 2003 and is China's earliest national, comprehensive and ongoing academic survey. The CGSS collects extensive data from community, family and individual levels of society. CGSS data is currently one of the most important sources for studying Chinese society and is widely used in research, education and government decision-making. The 2017 CGSS used a multistage stratified random sampling strategy to collect data at multiple levels of provinces, cities, counties, families and individuals by means of household surveys. The resultant sample included 12,582 persons from 28 provinces. Of these, 11,061 questionnaires were successfully completed, constituting a response rate of 87.9%.

We focused on respondents aged 23 to 65 years. We adopted a lower boundary of 23 years of age (i.e., born before 1994) to ensure that most respondents would have completed their educational training at the time of the interview. We adopted an upper boundary of 65 years of age (i.e., born after 1952) to ensure that the respondents were of working age and born after the founding of the People's Republic of China. Father's education (6.3%) and equivalized annual household income (8.6%) had the highest amounts of missing data in this subsample. We utilized a listwise deletion approach applied to the variables utilized in this study which led to discarding 15.9 % of the cases. Regarding missing data, we conducted sensitivity tests to compare the retained cases to dropped cases in regards to self-assessed mental health. We found no statistically significant difference between the two groups.

In light of the profound social transformations that the People's Republic of China experienced in the 1970s, we stratified our sample by age cohort, distinguishing between survey respondents aged 45 to 65 (born prior to 1972) from those aged 23 to 44 (born 1972 or later). The final working sample comprised 7733 respondents of whom 3356 were aged between 23 and 44 and 4377 were aged between 45 and 65.

We measured parental socioeconomic resources with father's educational attainment, mother's educational attainment and respondent's self-reported childhood social class. The parental educational attainment variables distinguished between (i) non-educated, (ii) elementary school, (iii) junior high school and (iv) technical school/senior high and higher. Compulsory education in contemporary China ranges from elementary school to junior high school but only came into force in 1986. Accordingly, we distinguished between elementary school and junior high school when coding father's and mother's education. To estimate self-reported childhood social class, respondents were asked, “Which social class did your family belong to when you were 14 years old?” Response categories ranged from 1 (lowest) to 10 (highest). We collapsed this ten-part variable into three categories, distinguishing between lower class (values 1 and 2), middle class (values 3 and 4) and higher class (values 5 to 10).

We measured personal socioeconomic resources with educational attainment and household income. Educational attainment distinguished between (i) non-educated, (ii) elementary school, (iii) junior high school, (iv) technical or senior high school, (v) junior college or higher and (vi) bachelor's degree or higher. Annual household income was measured in *Yuan*. We calculated logged equivalized annual household income by dividing annual household income by the square root of household size and then taking the log of this value.

We utilized self-assessed mental health as the dependent variable for these analyses. Self-rated health in general is considered to be an effective predictor of mortality and other functional limitations and has been used in many developed and developing countries. Here we focused on self-assessed anxiety/depression level in particular, the only measure of mental health and wellbeing in the 2017 CGSS. Respondents were asked “How often do you feel anxious and depressed?” with possible responses (i) always, (ii) often, (iii) sometimes, (iv) seldom and (v) never. We dichotomized this variable for use in multivariate binary logistic regression models by distinguishing between poor mental health (i and ii) and good mental health (iii, iv and v).

Control variables were age in years and its square, gender, marital status and hukou status. The hukou system (urban versus rural) is a household registration system in China that was first implemented in 1958 and limits opportunity for rural people in almost every sphere of social life including education, job opportunities, housing and health insurance (Liu, 2005). All of the variables utilized in the study are summarized in Table 1.

**Table 1:** Sample characteristics.

	Aged 23 to 44	Aged 45 to 65
	*n (%)*	*n (%)*
**Father's education**		
Non-educated	553 (16.48)	2086 (47.66)
Elementary school	1094 (32.60)	1538 (35.14)
Junior high school	923 (27.50)	414 (9.46)
Technical school/senior high and higher	786 (23.42)	339 (7.75)
**Mother's education**		
Non-educated	1037 (30.90)	3074 (70.23)
Elementary school	1130 (33.67)	930 (21.25)
Junior high school	681 (20.29)	220 (5.03)
Technical school/senior high and higher	508 (15.14)	153 (3.50)
**Respondent's education**		
Non-educated	103 (3.07)	537 (12.27)
Elementary school	392 (11.68)	1117 (25.52)
Junior high school	951 (28.34)	1457 (33.29)
Technical school/Senior high school	657 (19.58)	821 (18.76)
Junior college	450 (13.41)	267 (6.10)
Bachelor degree or higher	803 (23.93)	178 (4.07)
**Self-reported childhood social class**		
Lower	1087 (32.39)	2041 (46.63)
Middle	1239 (36.92)	1380 (31.53)
Higher	1030 (30.69)	956 (21.84)
**Hukou status**		
Rural	2085 (62.13)	2861 (65.36)
Urban	1271 (37.87)	1516 (34.64)
**Gender**		
Male	1578 (47.02)	2083 (47.59)
Female	1778 (52.98)	2294 (52.41)
**Marital Status**		
Unmarried	619 (18.44)	143 (3.27)
Married	2621 (78.10)	3835 (87.62)
Separated/divorced/bereaved	116 (3.46)	399 (9.11)
**Self-rated mental health**		
Very healthy/healthy/neutral	3098 (92.31)	3888 (88.83)
Unhealthy/very unhealthy	258 (7.69)	489 (11.17)
**Age in years**	mean = 34.2	mean = 54.9
	sd = 6.16	sd = 6.17
**Respondent logged equivalized income**	mean = 10.60	mean = 10.05
	sd = 1.09	sd = 1.16
**Total n**	3356	4377

The first stage of the analysis begins with a series of binary logistic regression models regressed on respondent education (bachelor's degree versus no bachelor's degree) followed by a series of OLS regression models regressed on respondent household income. This stage of analysis establishes the nature and extent of intergenerational reproduction in the two age groupings. The second stage of the study involves a series of binary logistic regression models regressed on self-self-assessed mental health. Where appropriate, we applied Wald tests (Stata command *testparm*) to determine whether a categorical variable makes a statistically significant contribution to a model. Where appropriate, we also applied the Karlson-Holm-Breen method (Stata command khb) of decomposing effects in non-linear probability models (Kohler et al., 2011) to investigate mediation in nested regression models. This method addresses the problem of residual variance in logit models in particular wherein changes in regression coefficients across nested models can reflect changes in the scaling of the dependent variable rather than mediation or confounding.

## 3 Results

### 3.1 Intergenerational reproduction

Table 2 summarizes binary logistic regression models regressed on respondent education (university degree or not). The first model includes father's and mother's education, controlling for age, square of age, gender, marital status and hukou status. The second model adds self-reported childhood social class to the first model. Model 1 in Table 2 indicates that both father's and mother's education are strongly and significantly associated with respondent education for both age groups (Wald test p < 0.001), with odds ratios comparing fathers with education level higher than technical school/senior high school to non-educated fathers of 5.036 (95% CI = 2.832-8.913) among respondents aged 23 to 44 and 3.324 (95% CI = 1.605-6.884) among respondents aged 45 to 65. The odds ratios comparing mothers with education level higher than technical school/senior high school to non-educated mothers are 3.783 (95% CI = 2.490-5.746) among respondents aged 23 to 44 and 5.155 (95% CI = 2.664-9.978) among respondents aged 45 to 65. Model 2 in Table 2 indicates that childhood social class is also significantly associated with respondent education in the younger group, with an odds ratio of 1.602 (95% CI = 1.205-2.131) comparing higher class to lower class. The KHB technique indicates that the coefficient comparing the highest and lowest education categories for the parents declined by 4.49% for father's education and 2.37% for mother's education upon controlling for childhood social class. Self-reported childhood social class is not significantly associated with respondent education among respondents aged between 45 and 65 (Wald test p > 0.1). The KHB technique indicates that the coefficients comparing the highest and lowest father's and mother's education categories declined by less than 5% upon controlling for childhood social class. These results indicate that parental education is strongly associated with their adult children's acquisition of university degrees for both younger and older respondents and that childhood social class and respondent university degree are somewhat more strongly associated among younger respondents. Only a small portion of the association between parental education and respondent education is explained by childhood social class in either age group.

**Table 2:** Binary logistic regressions on respondent education (university degree).

Aged 23 to 44	Model 1 *OR (95% CI)*	Model 2 *OR (95% CI)*
**Father's education**		
Non-educated (reference)	1.000	1.000
Elementary school	2.297** (1.340-3.938)	2.244** (1.307-3.853)
Junior high school	3.313*** (1.845-5.791)	3.115*** (1.779-5.452)
Technical school/senior high and higher	5.036*** (2.832-8.913)	4.711*** (2.656-8.356)
	*(Wald test p<0.001)*	*(Wald test p<0.001)*
**Mother's education**		
Non-educated (reference)	1.000	1.000
Elementary school	1.538* (1.084-2.182)	1.513* (1.065-2.150)
Junior high school	2.018*** (1.372- 2.970)	1.958** (1.327-2.889)
Technical school/senior high and higher	3.783*** (2.490-5.746)	3.654*** (2.391- 5.584)
	*(Wald test p<0.001)*	*(Wald test p<0.001)*
**Self-reported childhood social class**		
Lower (reference)	–	1.000
Middle	–	1.527** (1.154-2.020)
Higher	–	1.602** (1.205-2.131)
		*(Wald test p<0.01)*

**Aged 45 to 65**	**Model 1** *OR (95% CI)*	**Model 2** *OR (95% CI)*
**Father's education**		
Non-educated (reference)	1.000	1.000
Elementary school	2.041* (1.099-3.790)	1.987* (1.066-3.705)
Junior high school	1.540 (0.713-3.327)	1.487 (0.684-3.231)
Technical school/senior high and higher	3.324** (1.605-6.884)	3.215** (1.547-6.681)
	*(Wald test p<0.01)*	*(Wald test p<0.01)*
**Mother's education**		
Non-educated (reference)	1.000	1.000
Elementary school	2.309** (1.399-3.813)	2.247** (1.366-3.696)
Junior high school	3.026** (1.529- 5.987)	2.884** (1.461-5.694)
Technical school/senior high and higher	5.155*** (2.664-9.978)	4.861*** (2.536-9.317)
	*(Wald test p<0.001)*	*(Wald test p<0.001)*
**Self-reported childhood social class**		
Lower (reference)	–	1.000
Middle	–	1.398 (0.879-2.223)
Higher	–	1.429 (0.902-2.262)
		*(Wald test p>0.1)*

***p<0.001, **p<0.01, *p<0.05.

*Note: Each model controls for age, square of age, gender, marital status and hukou status.*

Table 3 provides the results of OLS regression models regressed on respondent equivalized household income. The first model includes father's and mother's education and control variables. Model 2 adds self-reported childhood social class to the first model and Model 3 adds respondent education to Model 2. Model 1 in Table 3 indicates that both father's and mother's education are significantly associated with respondent equivalized household income (Wald test p < 0.001). The coefficient for fathers having education level higher than technical/senior high school versus non-educated fathers is 0.472 (95% CI = 0.311-0. 632) for respondents aged 23 to 44 and 0.356 (95% CI = 0.176-0.536) for respondents aged 45 to 65. The coefficient for mothers having education level higher than technical/senior high school versus non-educated mothers is 0.628 (95% CI = 0.467-0.790) for respondents aged 23 to 44 and 0.523 (95% CI = 0.334-0.715) for respondents aged 45 to 65. Model 2 in Table 3 indicates that self-reported childhood social class is also significantly associated with respondent equivalized household income (Wald test p < 0.001) with coefficients of 0.441 (95% CI = 0.341-0.542) for the younger group and 0.412 (95% CI = 0.320-0.505) for the older group when comparing the higher and lower social class categories. Both parental education variables remain significantly associated with respondent household income after controlling for self-reported childhood social class in both age groups. The KHB technique indicates that the coefficients comparing the highest and lowest education categories declined by 13.04% for father's education and by 7.29% for mother's education in the younger age group upon controlling for childhood social class. In the older age group, the coefficient comparing the highest and lowest education categories declined by 10.92% for father's education and by 17.38% for mother's education upon controlling for childhood social class. Model 3 of Table 3 indicates that childhood social class retains a strong and significant association with respondent household income after controlling for respondent education attainment for both age groups. However, parents' education only retains a strong and significant association among the younger group but not the older group. The KHB technique indicates that the coefficient comparing the highest and lowest education categories declined by 58.91% (for fathers) and 46.18% (for mothers) in the younger age group and by 67.43% (for fathers) and 62.95% (for mothers) in the older age group upon controlling for respondent education. Also, the coefficient comparing the highest and lowest childhood social class categories declined by 25.43% in the younger age group and by 14.77% in the older age group upon controlling for respondent education. Lastly, respondent education is significantly associated (Wald test p < 0.001) with respondent household income after controlling for parental resources in both age groups.

**Table 3:** OLS regressions on respondent equivalized household income.

Aged 23 to 44	Model 1 *b (95% CI)*	Model 2 *b (95% CI)*	Model 3 *b (95% CI)*
**Father's education**			
Non-educated	(reference)	(reference)	(reference)
Elementary school	0.151* (0.023-0.279)	0.122 (-0.004-0.249)	0.013 (-0.109-0.134)
Junior high school	0.383*** (0.242-0.523)	0.326*** (0.188-0.463)	0.122 (-0.010-0.255)
Technical school/senior high and higher	0.472*** (0.311-0. 632)	0.409*** (0.251-0.567)	0.169* (0.021-0.317)
	*(Wald test p<0.001)*	*(Wald test p<0.001)*	*(Wald test p<0.05)*
**Mother's education**			
Non-educated	(reference)	(reference)	(reference)
Elementary school	0.257*** (0.150-0.363)	0.243*** (0.139-0.348)	0.142** (0.047-0.237)
Junior high school	0.365*** (0.235-0.495)	0.339*** (0.211-0.467)	0.148* (0.028-0.267)
Technical school/senior high and higher	0.628*** (0.467-0.790)	0.579*** (0.420-0.738)	0.310*** (0.164-0.456)
	*(Wald test p<0.001)*	*(Wald test p<0.001)*	*(Wald test p<0.001)*
**Self-reported childhood social class**			
Lower	–	(reference)	(reference)
Middle	–	0.249*** (0.157-0.341)	0.133** (0.049-0.217)
Higher	–	0.441*** (0.341-0.542)	0.331*** (0.240-0.421)
		*(Wald test p<0.001)*	*(Wald test p<0.001)*
**Respondent's education**			
Non-educated	–	–	(reference)
Elementary school	–	–	0.016 (-0.243-0.275)
Junior high school	–	–	0.311* (0.066-0.556)
Technical school/Senior high	–	–	0.743*** (0.492-0.994)
Junior college	–	–	1.061*** (0.798-1.324)
Bachelor's degree or higher	–	–	1.342*** (1.084-1.600)
			*(Wald test p<0.001)*

Aged 45 to 65	Model 1 *b (95% CI)*	Model 2 *b (95% CI)*	Model 3 *b (95% CI)*
**Father's education**			
Non-educated (reference)	1.000	1.000	1.000
Elementary school	0.178*** (0. 091-0.265)	0.153** (0.067-0.240)	0.076 (-0.008-0.160)
Junior high school	0.187** (0.049-0.325)	0.163* (0.027-0.299)	0.041 (-0.087-0.169)
Technical school/senior high and higher	0.356*** (0.176-0.536)	0.317** (0.136-0.498)	0.098 (-0.077-0.273)
	*(Wald test p<0.001)*	*(Wald test p<0.001)*	*(Wald test p>0.05)*
**Mother's education**			
Non-educated (reference)	1.000	1.000	1.000
Elementary school	0.226*** (0.126-0.325)	0.188*** (0.091-0.285)	0.081 (-0.011-0.173)
Junior high school	0.333*** (0.173-0.493)	0.266** (0.108-0.423)	0.119 (-0.025-0.263)
Technical school/senior high and higher	0.523*** (0.334-0.715)	0.434*** (0.245-0.623)	0.157 (-0.017-0.331)
	*(Wald test p<0.001)*	*(Wald test p<0.001)*	*(Wald test p>0.05)*
**Self-reported childhood social class**			
Lower (reference)	–	1.000	1.000
Middle	–	0.275*** (0.194-0.356)	0.216*** (0.138-0.294)
Higher	–	0.412*** (0.320-0.505)	0.345*** (0.255-0.435)
		*(Wald test p<0.001)*	*(Wald test p<0.001)*
**Respondent's education**			
Non-educated (reference)	–	–	1.000
Elementary school	–	–	0.137* (0.001-0.274)
Junior high school	–	–	0.362*** (0.225-0.498)
Technical school/Senior high	–	–	0.607*** (0.455-0.760)
Junior college	–	–	1.095*** (0.926-1.264)
Bachelor's degree or higher	–	–	1.371*** (1.185-1.558)
			*(Wald test p<0.001)*

***p<0.001, **p<0.01, *p<0.05.

*Note: Each model controls for age, square of age, gender, marital status and hukou status.*

### 3.2 Intergenerational reproduction and self-rated anxiety/depression level

Table 4 presents binary logistic regression models on poor self-assessed mental health. Model 1 includes father's and mother's education and control variables. Model 2 adds self-reported childhood social class to the first model, Model 3 adds respondent education to Model 2 and Model 4 adds respondent equivalized family income to Model 3. Model 1 indicates that father's and mother's educational attainment are not significantly associated with self-assessed mental health for both younger and older respondents. Model 2 indicates that childhood social class manifests a significant association with self-assessed mental health among both younger and older respondents and, surprisingly, the middle class is most divergent from the upper class with odds ratios of 0.507 (95% CI = 0.355-0.725) among younger respondents and 0.623 (95% CI = 0.478-0.811) among older respondents. The odds ratio comparing higher class to lower class is 0.658 (95% CI = 0.447-0.970) among younger respondents and 0.728 (95% CI = 0.532-0.996) among older respondents. Model 3 and Model 4 also indicate that, for both age groups, these odds ratios are somewhat reduced by controlling for respondent education and household income. In addition, Models 3 and 4 show that respondent education attainment is significantly associated (Wald test p < 0.01) with self-assessed mental health in the younger age group while respondent household income is significantly associated (Wald test p < 0.001) with self-assessed mental health in the older age group after controlling for parental resources. Specifically, the KHB technique indicates that the upper-class odds ratio versus the lower-class odds ratio was reduced by 16.21% among younger respondents and by 61.21% among older respondents, with respondent education largely responsible for the decline among younger respondents and respondent household income largely responsible for the decline among older respondents.

**Table 4:** Binary logistic regressions on fair/poor self-rated mental health.

Aged 23 to 44	Model 1 *OR (95% CI)*	Model 2 *OR (95% CI)*	Model 3 *OR (95% CI)*	Model 4 *OR (95% CI)*
**Father's education**				
Non-educated (reference)	1.000	1.000	1.000	1.000
Elementary school	0.937 (0.601-1.459)	0.948 (0.605-1.485)	1.072 (0.681-1.690)	1.073 (0.682-1.688)
Junior high school	0.825 (0.487-1.398)	0.888 (0.522-1.511)	1.061 (0.612-1.840)	1.072 (0.618-1.861)
Technical school/senior high and higher	0.954 (0.540-1.686)	1.034 (0.585-1.828)	1.218 (0.674-2.204)	1.230 (0.681-2.223)
	*(Wald test p>0.1)*	*(Wald test p>0.1)*	*(Wald test p>0.1)*	*(Wald test p>0.1)*
**Mother's education**				
Non-educated (reference)	1.000	1.000	1.000	1.000
Elementary school	0.843 (0.569-1.250)	0.858 (0.579-1.273)	0.958 (0.647-1. 417)	0.970 (0.656-1.434)
Junior high school	1.123 (0.666-1.892)	1.144 (0.680-1.926)	1.364 (0. 810-2.296)	1.383 (0.821-2.319)
Technical school/senior high and higher	1.024 (0.551-1.905)	1.022 (0.542-1.926)	1.165 (0.611-2.224)	1.195 (0.629-2.269)
	*(Wald test p>0.1)*	*(Wald test p>0.1)*	*(Wald test p>0.1)*	*(Wald test p>0.1)*
**Self-reported childhood social class**				
Lower (reference)	–	1.000	1.000	1.000
Middle	–	0.507*** (0.355-0.725)	0.546** (0.380-0.787)	0.556** (0.384-0.805)
Higher	–	0.658* (0.447-0.970)	0.708 (0.475-1.058)	0.733 (0.486-1.106)
		*(Wald test p<0.001)*	*(Wald test p<0.01)*	*(Wald test p<0.01)*
**Respondent's education**				
Non-educated (reference)	–	–	1.000	1.000
Elementary school	–	–	0.771 (0.361-1.649)	0.775 (0.364-1.651)
Junior high school	–	–	0.409* (0.193-0.868)	0.424* (0.201-0.894)
Technical school/Senior high	–	–	0.375* (0.167-0.841)	0.406* (0.182-0.905)
Junior college	–	–	0.221** (0.093-0.526)	0.247** (0.106-0.577)
Bachelor's degree or higher	–	–	0.367* (0.159-0.848)	0.422* (0.183-0.970)
			*(Wald test p<0.01)*	*(Wald test p<0.01)*
**Respondent's family income**				0.909 (0.777-1.063)

Aged 45 to 65	Model 1 *OR (95% CI)*	Model 2 *OR (95% CI)*	Model 3 *OR (95% CI)*	Model 4 *OR (95% CI)*
**Father's education**				
Non-educated (reference)	1.000	1.000	1.000	1.000
Elementary school	0.902 (0.682-1.191)	0.918 (0.693-1.215)	0.966 (0.725-1.288)	0.995 (0.743-1.333)
Junior high school	0.963 (0.604-1.534)	0.968 (0.610-1.538)	1.061 (0.663-1.697)	1.076 (0.672-1.724)
Technical school/senior high and higher	1.002 (0.547-1.835)	1.029 (0.558-1.900)	1.188 (0.640-2.204)	1.261 (0.668-2.377)
	*(Wald test p>0.1)*	*(Wald test p>0.1)*	*(Wald test p>0.1)*	*(Wald test p>0.1)*
**Mother's education**				
Non-educated (reference)	1.000	1.000	1.000	1.000
Elementary school	1.010 (0.726-1.407)	1.044 (0.747-1.458)	1.117 (0.799-1.560)	1.143 (0.814-1.607)
Junior high school	0.729 (0.361-1.474)	0.757 (0.376-1. 524)	0.816 (0.403-1.654)	0.853 (0.418-1.740)
Technical school/senior high and higher	1.356 (0.600-3.064)	1.424 (0.619-3.273)	1.614 (0.676-3.856)	1.711 (0.716-4.084)
	*(Wald test p>0.1)*	*(Wald test p>0.1)*	*(Wald test p>0.1)*	*(Wald test p>0.1)*
**Self-reported childhood social class**				
Lower (reference)	–	1.000	1.000	1.000
Middle	–	0.623*** (0.478-0.811)	0.646** (0.495-0.843)	0.712* (0.543-0.933)
Higher	–	0.728* (0.532-0.996)	0.763 (0.557-1.044)	0.895 (0.651-1.231)
		*(Wald test p<0.01)*	*(Wald test p<0.01)*	*(Wald test p<0.05)*
**Respondent's education**				
Non-educated (reference)	–	–	1.000	1.000
Elementary school	–	–	0.895 (0.640-1.251)	0.955 (0.683-1.336)
Junior high school	–	–	0.782 (0.547-1.117)	0.919 (0.642-1.315)
Technical school/senior high	–	–	0.634* (0.409-0.984)	0.825 (0.531-1.282)
Junior college	–	–	0.314** (0.145-0.684)	0.520 (0.236-1.147)
Bachelor's degree or higher	–	–	0.609 (0.261-1.419)	1.146 (0.484-2.712)
			*(Wald test p<0.1)*	*(Wald test p>0.1)*
**Respondent's family income**	–	–	–	0.644*** (0.578-0.717)

***p<0.001, **p<0.01, *p<0.05.

*Note: Each model controls for age, square of age, gender, marital status and hukou status.*

## 4 Discussion

Consistent with the intergenerational elasticities in education and income unveiled by the World Bank (Fan et al., 2019; Mobility Global Database on Intergenerational, 2018), we discovered strong elasticities between parental socioeconomic resources and respondent socioeconomic status. Specifically, after controlling for one another, parental education attainment and childhood social class were both associated with respondent education for both age groups, indicating that parental education appears to play a large role in shaping respondents' education and income for both cohorts. This suggests that parents with higher levels of education may be better equipped to help their children succeed in China's competitive educational environment, particularly in the group of people born between 1952 and 1972 who came of age during the planned economy period of the People's Republic of China when the majority of parents wanted their children to quit school and work to help support the family. Parents with higher levels of education may be more likely to encourage their children to continue their education and eventually pursue careers that provide higher incomes. Given this finding, it is possible that the Chinese economic reform of the 1970s, which boosted personal prosperity and economic progress (Hong & Zhao, 2015; Zhao, 2016), also added another barrier to upward social mobility.

In addition, our findings corroborate the idea that parental education and childhood social class both significantly impact individual family earnings, with childhood social class explaining only a modest portion of the association. Personal education statistically explained a substantial proportion of the association between parental education and respondent household income, slightly more so in the older age group, whereas the relationship between childhood social class and respondent household income was only attenuated by 25% in the younger cohort and 15% in the older cohort after controlling for personal education. The former result suggests that, in China, helping children succeed in the competitive education system pays off in the form of higher salaries in adulthood. According to the latter finding, participation in higher education may not be the only important pathway through which socioeconomic standing is passed down through the generations. In China, the transmission of social class may occur through monetary inheritance (Zhu, 2018) as well as parents utilizing strong social ties to help their children acquire better jobs (Bian, 1997). As Hong and Zhao (2015) have previously concluded, it appears that economic capital and cultural capital both function to sustain the so-called crystallization of social classes in China.

Differences in the impact of mother's and father's educational attainment on their children's socioeconomic status are also noteworthy. An assumption commonly made in mainstream stratification research is that the father's social resources most accurately reflect the socioeconomic status of a family (Goldthorpe, 1983; Korupp et al., 2002). In this dataset, the mother's education attainment is significantly lower than the father's educational attainment for both age groups. However, we still find meaningful effects of mother's education on children's socioeconomic status. Indeed, the educational achievement of mothers has a significantly stronger impact on the educational attainment of their children among respondents who were born prior to 1972. In regards to family earnings, mother's educational achievement plays a stronger role than father's educational attainment for both cohorts. One potential explanation for this is that, despite having fewer socioeconomic resources than fathers, mothers in China exhibit a higher level of dedication to parenting, potentially resulting in a greater impact on their children's development. Consistent with this hypothesis, research in Nepal reveals a strong and direct relationship between children's health outcomes and mother's educational attainment but not with father's educational attainment (Karki Nepal 2018). Contrarily, research from The Netherlands, West Germany and the USA revealed a equal influence of mother's and father's education on children's education attainment (Korupp et al., 2002). Our research indicates that it is important to focus on both father's and mother's educational achievement in intergenerational transmission studies, even in societies like China that are commonly perceived as being more patriarchal.

Given the intergenerational elasticity of socioeconomic resources, we find some evidence for the idea that intergenerational reproduction influences adult self-assessed mental health. Parental education is not significantly relevant for self-assessed mental health for either age group. However, the previously observed elasticities between childhood social class and respondent socioeconomic resources are germane to self-assessed mental health in both age groups. We discovered, to our surprise, that respondents who reported being middle class rather than higher class as children are the least likely to report poor mental health. Personal education and household income attenuated some of the associations between parental socioeconomic resources and poor mental health. In the younger group, less than a fifth of the association between childhood social class and self-assessed mental health was explained by personal socioeconomic resources, whereas in the older group personal socioeconomic resources explained a large portion of the association. These results echo the cumulative exposure scenario of intergenerational transmission of mental health which suggests that both parental and personal socioeconomic resources play a part in shaping adulthood mental health (Berkman, 2009). It is also worth noting that, although both personal education and household income apparently matter for self-assessed mental health, they matter differently for younger and older respondents. For younger respondents, it is their education level that is more germane to poor mental health whereas for the older group it is their household income that mainly explains their mental wellbeing. Our results also suggest that parental resources have a strong influence on the mental health of both younger individuals and older individuals whereas for the older group mental health is more reflective of the resources they currently possess than those previously held by their parents.

Previous research in China has illuminated the importance of intergenerational reproduction in fostering the good physical health of Chinese adults, especially for those who grew up after the Chinese economic reforms of the 1970s (Zhang & Veenstra, 2022). In this study, we uncovered evidence that social class, and specifically childhood social class, manifests an independent connection with current mental health, especially for people born after 1972. The limitations of this study help to point the way for future research in this field by providing some general guidelines. First, a measure of self-assessed feelings of anxiety or depression conflates anxiety and depression which can be quite different psychological experiences. Second, the measure of parental economic resources we utilized in our study is not robust. We employed a subjective and retrospective measure of self-rated childhood social class that is not a direct and dependable measure of economic resources. Future research in this field would benefit from having access to more objective measures of the socioeconomic status of children's families. Finally, due to the cross-sectional nature of the data used in this investigation, we are not in a position to definitively determine causal directionality. In this area of inquiry, longitudinal data that incorporates information from a number of different generations would be particularly valuable.

## 5 Conclusion

Using data from the 2017 Chinese General Social Survey, we documented significant elasticities between respondents' socioeconomic status and their parents' socioeconomic status. We also found reason to believe that intergenerational reproduction influences adult self-assessed mental health, as associations between parental socioeconomic status and their adult children's self-assessed mental health were statistically explained to some extent by the children's own socioeconomic status. However, these correlations were moderated by age cohort. Educational attainment explained a sizeable portion of the association between parental socioeconomic status and mental health in the younger age group, whereas household income explained a sizeable portion of the association in the older age group. In general, parental socioeconomic status appears to have a higher influence on the mental health of younger people who grew up after the Chinese economic reform of the 1970s.

Nowadays, the phrase “寒门难出贵子(It's hard to have a noble son from a poor family)” is popular in Chinese news and social media (Yang, 2017). Our research provides further insight into the nature of intergenerational socioeconomic reproduction in China. It is important to carefully evaluate and address the barriers that impede educational access and hamper the achievement of prestigious occupational roles by disadvantaged young people. Our findings suggest that implementing steps to address these concerns could potentially reduce the influence of intergenerational socioeconomic reproduction on mental health.

## Ethical statement

Please note that the manuscript has not been published elsewhere and is not under submission elsewhere. Also note that there is no conflict of interest to report and that both authors have reviewed the submitted manuscript and approve the manuscript for submission.

The study was approved by the Behavioural Research Board at the University of British Columbia.

## CRediT authorship contribution statement

**Xueqing Zhang:** Writing – review & editing, Writing – original draft, Methodology, Investigation, Formal analysis, Data curation, Conceptualization. **Gerry Veenstra:** Writing – review & editing, Supervision, Formal analysis.

## Data Availability

Data will be made available on request.
